# Correlations of Ventricular Enlargement with Rheologically Active Surfactant Proteins in Cerebrospinal Fluid

**DOI:** 10.3389/fnagi.2016.00324

**Published:** 2017-01-04

**Authors:** Stefan Schob, Alexander Weiß, Julia Dieckow, Cindy Richter, Mandy Pirlich, Peter Voigt, Alexey Surov, Karl-Titus Hoffmann, Ulf Quaeschling, Matthias Preuß

**Affiliations:** ^1^Department of Neuroradiology, Leipzig UniversityLeipzig, Germany; ^2^Department of Ophthalmology, Leipzig UniversityLeipzig, Germany; ^3^Institute of Anatomy, Leipzig UniversityLeipzig, Germany; ^4^Department of Neurology, Leipzig UniversityLeipzig, Germany; ^5^Department of Diagnostic and Interventional Radiology, Leipzig University HospitalLeipzig, Germany; ^6^Department of Neurosurgery, Leipzig UniversityLeipzig, Germany

**Keywords:** MRI, surfactant proteins, CSF, ventricular enlargement, Evans’ index

## Abstract

**Purpose**: Surfactant proteins (SPs) are involved in the regulation of rheological properties of body fluids. Concentrations of SPs are altered in the cerebrospinal fluid (CSF) of hydrocephalus patients. The common hallmark of hydrocephalus is enlargement of the brain ventricles. The relationship of both phenomena has not yet been investigated. The aim of this study was to evaluate the association between SP concentrations in the CSF and enlargement of the brain ventricles.

**Procedures**: Ninty-six individuals (41 healthy subjects and 55 hydrocephalus patients) were included in this retrospective analysis. CSF specimens were analyzed for SP-A, SP-B, SP-C and SP-D concentrations by use of enzyme linked immunosorbent assays (ELISA). Ventricular enlargement was quantified in T2 weighted (T2w) magnetic resonance imaging (MRI) sections using an uni-dimensional (Evans’ Index) and a two-dimensional approach (lateral ventricles area index, LVAI).

**Results**: CSF-SP concentrations (mean ± standard deviation in ng/ml) were as follows: SP-A 0.71 ± 0.58, SP-B 0.18 ± 0.43, SP-C 0.89 ± 0.77 and SP-D 7.4 ± 5.4. Calculated values of Evans’ Index were 0.37 ± 0.11, a calculation of LVAI resulted in 0.18 ± 0.15 (each mean ± standard deviation). Significant correlations were identified for Evans’ Index with SP-A (*r* = 0.388, *p* < 0.001) and SP-C (*r* = 0.392, *p* < 0.001), LVAI with SP-A (*r* = 0.352, *p* = 0.001), SP-C (*r* = 0.471, *p* < 0.001) and SP-D (*r* = 0.233, *p* = 0.025). Furthermore, SP-C showed a clear inverse correlation with age (*r* = −0.357, *p* = 0.011).

**Conclusion**: The present study confirmed significant correlations between SPs A, C and D in the CSF with enlargement of the inner CSF spaces. In conclusion, SPs clearly play an important role for CSF rheology. CSF rheology is profoundly altered in hydrocephalic diseases, however, diagnosis and therapy of hydrocephalic conditions are still almost exclusively based on ventricular enlargement. Until now it was unclear, whether the stage of the disease, as represented by the extent of ventricular dilatation, is somehow related to the changes of SP levels in the CSF. Our study is the first to provide evidence that increasing ventriculomegaly is accompanied by enhanced changes of rheologically active compounds in the CSF and therefore introduces completely new aspects for hydrocephalus testing and conservative therapeutic approaches.

## Introduction

Surfactant proteins (SPs) are a heterogeneous group of proteins being present at various sites in the body (Snegovskikh et al., 2011; Yadav et al., 2011; Hallman, 2013). SPs have been described initially in the respiratory system (Phizackerley et al., 1979). Two functionally different SP-fractions exist, the collectin type SPs (SP-A and SP-D) and the hydrophobic SPs (SP-B and SP-C). Collectin type SPs are soluble pattern-recognition proteins of the innate immunity, functionally resembling the antibodies of the adaptive immune system (Palaniyar, 2010). They mostly bear—among other cellular and humoral components—responsibility for host defense and alleviation of detrimental immune responses in the lungs (Wright, 2005; Hallman, 2013). Hydrophobic SPs (SP-B and SP-C) decrease surface tension of the alveolar fluid by direct interaction with phospholipids, thus facilitating the mechanism of respiration (Weaver, 1997; Johansson, 1998; Schürch et al., 2010). However, both fractions are essential for surfactant functionality (Chroneos et al., 2010). The physiological significance of SPs is underlined by the severity of the natural course of conditions secondary to surfactant deficiency or dysfunction, for example respiratory distress syndrome of the (prematurely) newborn. In premature infants, type II epithelial cells, which are the main source of pulmonary surfactant, are not yet fully differentiated and therefore cannot produce sufficient amounts of the rheologically active compund. This condition results in RDS of the premature infant (Whitsett, 2014). Until 1970, RDS was a mostly lethal condition leading to respiratory failure regardless of the degree of prematurity. Animal models of RDS provided insight into surfactant physiology and revealed the curative potential of intratracheal surfactant replacement therapy (Enhörning and Robertson, 1972). As a consequence of this, surfactant replacement was succesfully introduced as the gold standard therapy of RDS, which significantly reduced mortality of the disease (Kendig et al., 1991).

Besides the lungs, SPs are further present at numerous barriers between distinct physiological compartments, for example in the gingiva epithelium of the oral cavity (Schicht et al., 2015), in the endometrium adjacent to the uterine cavity (Yadav et al., 2011) and in the testes corresponding the anatomical location of the blood-testis-barrier (Beileke et al., 2015). Considering the well investigated roles of SPs at the interface between tissue and avleolar fluid in the lungs and their remarkable expression pattern at other interfaces in the body, it can be assumed that SPs generally have a specific scope of functions at physiological boundaries.

In addition to the previously given interfaces, SPs are inherent proteins of the central nervous system (CNS) being abundant at the sites of the blood-brain barrier (BBB) and blood-CSF barrier (BSCFB; Schob et al., 2013). SP-A and SP-C appear in the CNS already during embryonic development, resembling the appearance pattern in the lungs, whereas SP-B and SP-D do not appear in the CNS until birth (Schob et al., 2016a). On a cellular level SPs of the CNS are produced within astrocytic feet processes surrounding parenchymal vessels, in the choroid plexus and by ventricular ependymal cells to finally be released into the CSF (Schob et al., 2016a). With reference to the well known effects of SPs on the physicochemical properties of the alveolar fluid, an important role of SPs for CSF rheology is presumable. This hypothesis is corroborated by a recent work, which demonstrated that SP concentrations in CSF are significantly altered in patients suffering from hydrocephalic conditions (Schob et al., 2016b). Hydrocephalus is one of the most challenging neurosurgical conditions to treat; for the past centuries, different pathophysiological concepts and discoveries lead to a yet incomplete understanding of the genesis of this entity (Preuss et al., 2013). Diagnostics and therapy until today still focus on ventricular enlargement; however, underlying pathomechanisms and counterregulatory processes are not sufficiently known (Preuss et al., 2013). To better understand hydrocephalic conditions on a molecular level and develop new, targeted therapeutic strategies, further insight in the relationship between disease-related morphological changes of the inner CSF spaces and alterations of rheologically active SPs in the CSF is elementary.

Therefore the aim of the present study was to use reliable, magnetic resonance imaging (MRI)-derived measurements (Evans’ Index (Ragan et al., 2015) and the newly established lateral ventricles area index (LVAI), reflecting enlargement of the brain ventricles, to investigate the association between CSF concentrations of rheologically important SPs and dilatation of the inner CSF spaces.

## Materials and Methods

This is an institutional review board (Ethikkommission Universität Leipzig Az 330-13-18112013) approved study with retrospective design.

### Patients

#### Inclusion Criteria

Patients were included in the hydrocephalus group if the diagnostic criteria for aqueductal stenosis (AQS), acute hydrocephalus (AH) or normal pressure hydrocephalus (NPH) were met. Patients were included in the control group if evidence of neurological disorders (clinical, neuroimaging and CSF examination) was absent. To be included in either group, MRI and CSF samples had to be available in addition to the clinical data.

If signs of infection or other inflammatory or autoimmune diseases were present, the patient was not included in the study. Also, evidence for blood brain barrier breakdown with leakage of serum proteins into the CSF was an exclusion criterium for both groups.

A total of 96 individuals was examined. All patients or caregivers gave their informed consent for the scientific use of CSF-samples, clinical and radiological data. Informed consent was given in writing. CSF specimens of 41 subjects without evidence of neurological pathologies were used as a control group. Those specimens were obtained during a diagnostic workup that necessitated CSF examination after lumbar puncture (e.g., exclusion of subarachnoid hemorrhage, demyelinating disease, meningitis). Furthermore, CSF samples were obtained from 55 patients finally diagnosed with hydrocephalus in the context of their diagnostic workup.

Hydrocephalus patients were categorized into the following subgroups after the review of patient records and brain imaging: AQS, AH (excluding AQS) and NPH. Twenty-one patients with narrowed or obstructed mesencephalic aqueduct and corresponding enlargement of the lateral and III. ventricles in cranial imaging were classified as AQS (Cinalli et al., 2011; Rodis et al., 2016). Nineteen patients exhibiting Hakim’s triad (gait disturbance, urinary incontinence, dementia) and morphological alterations in cranial MRI (cMRI) typical for NPH (ventriculomegaly and tight convexity sulci in combination with enlarged Sylvian fissures) were included after standardized clinical (Mahr et al., 2016) and radiological examination (Weerakkody et al., 2011; Preuss et al., 2013; Lieb et al., 2015). The group of AH patients consisted of 15 individuals and was a heterogenous group with AH of different pathophysiologies: posthemorrhagic, postinfectious and idiopathic hydrocephalus without radiological proof of occlusion of ventricular CSF outflow (formerly classified as communicating hydrocephalus). All AH patients presented signs of elevated intracranial pressure (Freeman, 2015). Table 1A gives an overview of the demographic data of hydrocephalic patients and the control group. Table 1B summarizes the demographical data of the hydrocephalic subgroups.

**Table 1A:** **Synopsis of demographic data of hydrocephalic patients vs. controls**.

	Control group	Hydrocephalic patients
*n*	41	55
Age in years	41.4	38.3
(mean, range)	2–84	<1–84
Sex (female/male)	17/14	27/28

**Table 1B:** **Synopsis of demographic data of investigated hydrocephalus subgroups**.

	AH	AQS	NPH
*n*	15	21	19
Age in years	25	21.2	67.7
(mean, range)	<1–75	<1–65	31–84
Sex (female/male)	9/6	12/9	6/13

*AH, acute hydrocephalus; AQS, aqueductal stenosis; NPH, normal pressure hydrocephalus*.

### Determination of Surfactant Protein Concentrations in CSF

SP concentrations were quantified using enzyme-linked immunosorbent assays (ELISA) according to the manufacturer’s manual. Commercially available ELISA kits (USCN, Wuhan, China) were used for quantification of SP-A (E90890Hu, ELISA Kit for Surfactant Associated Protein A), SP-B (E91622Hu, ELISA Kit for Surfactant Associated Protein B), SP-C (E91623Hu, ELISA Kit for Surfactant Associated Protein C) and SP-D (E91039Hu, ELISA Kit for Surfactant-Associated Protein D) in CSF samples. The analysis was performed using a microplate spectrophotometer (ELISA-reader) at a wavelength of 450 nm and a reference wavelength of 405 nm for absorbance measuring. Surfactant protein concentrations in ng/ml CSF were calculated by comparison between standard series and the determined values of antigen concentration (protein concentration) according to the manufacturer’s manual.

### Magnetic Resonance Imaging

In all patients cMRI was performed by using either a 1.5 T device (Magnetom Symphony, Siemens/Germany; Intera, Philips/Netherlands or Achieva, Philips/Netherlands) or 3 T device (Trio Tim, Siemens/Germany). The imaging protocols differed between the investigated individuals depending on the initially suspected diagnosis. All imaging protocols included an axial and sagittal T2 weighted (T2w) turbo spin echo sequence (TSE) as minimal imaging requirements, being necessary for evaluation of Evans’ Index/LVAI and identification of AQS or AH.

### Determination of Ventricular Enlargement—Evaluation of Evans’ Index and LVAI

Image analysis was performed by a neuroradiologist (UQ) with 15 years of radiological experience.

The Evans’ Index was determined using OsiriX lite software as follows: maximal width of the ventricular frontal horns divided by the maximal transverse width of the cranium (Evans, 1942; Ambarki et al., 2010).

**Figure 1:**
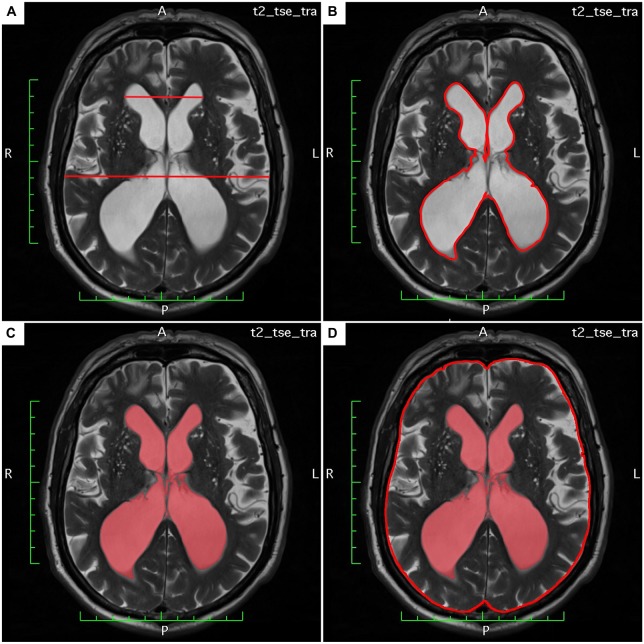
**(A–D)** Demonstrates estimation of Evan’s Index and lateral ventricles area index (LVAI) on axial T2 weighted (T2w) magnetic resonance imaging (MRI) sections.

The LVAI was estimated as follows: the axial section showing the maximal area of the lateral ventricles (frontal and occipital horns) was identified. The area of both frontal and occipital horns—the maximal lateral ventricular area—was quantified within this section using the area measurement function in OsiriX lite. The total intracranial (intradural) area in the same axial section was calculated, respectively. Finally, the index was calculated as follows: maximal lateral ventricular area divided by the corresponding intradural area. Figures 1A–D exemplarily demonstrate estimation of Evans’ Index and LVAI.

### Statistical Analysis

For statistical analysis the SPSS statistical software package was used (SPSS 23, SPSS Inc., Chicago IL, USA). Collected data was evaluated by means of descriptive statistics (mean, standard deviation, range). Comparison of CSF-SP concentrations, Evans’ Index and LVAI between hydrocephalic patients and controls as well as between the hydrocephalic subsets (acute hydrocephalic conditions (AQS + AH) and NPH) and controls were performed by means of ANOVA and—for the latter—Dunnett’s *T*-test as a *post hoc* algorithm. For Dunnett’s test the control group served as reference group for the hydrocephalic subsets. *p-values* < 0.05 were taken to indicate statistical significance in all instances. Spearman’s correlation coefficient was used to analyze the association between Evans’ Index, LVAI, age and CSF-SP concentrations.

## Results

The acquired data was analyzed in three subsequent steps: (i) CSF-SP concentrations and indices of ventricular dilatation (Evans’ Index and LVAI) were determined for the whole collective in order to elucidate whether there is a general association of SP concentration and ventricular enlargement. (ii) Calculations were performed once more after separation into two subgroups—all hydrocephalic patients and healthy controls—to corroborate the gathered findings by comparing changes in SP concentrations between individuals with and without reliably proven ventricular enlargement. (iii) The hydrocephalic subgroup was further devided into etiological subsets—acute hydrocephalic conditions (AQS plus AH) vs. NPH—in order to challenge the previously reported exceptions of the rather chronic hydrocephalic disease NPH regarding the differences of SP concentration in the CSF.

### Overall Collective

The calculated CSF-SP concentrations (mean ± standard deviation) over all investigated subjects were as follows: SP-A = 0.71 ± 0.58 ng/ml, SP-B = 0.18 ± 0.43 ng/ml, SP-C = 0.89 ± 0.77 ng/ml and SP-D = 7.4 ± 5.4 ng/ml (Table 2). Analysis of cMRI of all subjects revealed the following results (mean values ± standard deviation): Evans’ Index = 0.37 ± 0.11, LVAI = 0.18 ± 0.15. Table 4 compares CSF-SP concentrations and indices of ventricular enlargement between hydrocephalic patients and the control group.

**Table 2:** **Overview of CSF-SP concentrations and measures of ventricular enlargement (Evans’ Index, lateral ventricles area index (LVAI) in all investigated individuals**.

	Mean ± SD	Range
SP-A (ng/ml)	0.71 ± 0.58	0.03-2.34
SP-B (ng/ml)	0.18 ± 0.43	0.0−1.92
SP-C (ng/ml)	0.89 ± 0.77	0.10−5.18
SP-D (ng/ml)	7.4 ± 5.4	0.00−38.03
Evans’ Index	0.37 ± 0.11	0.09−0.73
LVAI	0.18 ± 0.15	0.01−0.79

**Figure 2:**
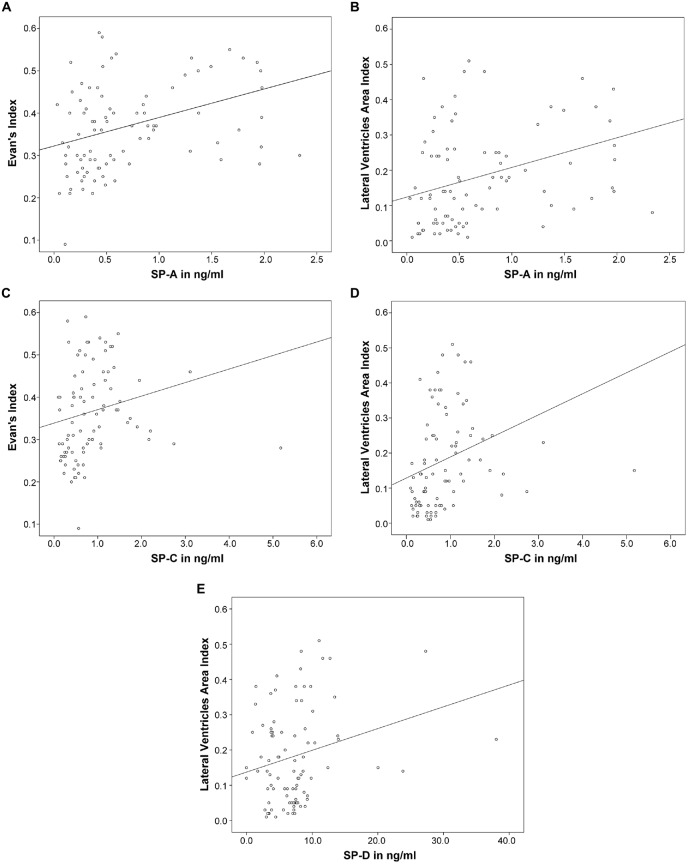
**(A–E)** Graphically demonstrates identified correlations between measures of ventricular enlargement (Evans’ Index and LVAI) and cerebrospinal fluid (CSF)-Surfactant protein (SP) concentrations (SP-A, SP-C and SP-D).

Subsequently, correlation analysis between CSF-SP concentrations and cMRI derived measures of ventricular enlargement (Evans’ Index, LVAI) was performed. As shown in Table 3; Figures 2A–D, SP-A and SP-C correlated in a statistically significant manner with Evans’ Index and LVAI (SP-A + Evans’ Index: *r* = 0.388, *p* < 0.001, SP-A + LVAI: *r* = 0.352; SP-C + Evans’ Index: *r* = 0.392, *p* < 0.001 SP-C + LVAI: *r* = 0.471, *p* < 0.001). However, correlation of SP-C was stronger with LVAI compared to Evans’ Index, as shown by the higher correlation coefficient (*r*). Interestingly, SP-D showed a statistically significant correlation with LVAI (*r* = 0.233, *p* = 0.025, graphically demonstrated in Figure 2E), but not with Evans’ Index. SP-B did not show any correlation with either index.

**Table 3:** **Results of the correlation analysis between measures of ventricular enlargement (Evans’ Index, LVAI) and CSF-SP levels of all investigated individuals**.

	SP-A	SP-B	SP-C	SP-D
Evans’ Index	*r* = 0.388	*r* = 0.038	*r* = 0.392	*r* = 0.168
	*p* = <0.001	*p* = 0.722	*p* = <0.001	*p* = 0.107
LVAI	*r* = 0.352	*r* = 0.041	*r* = 0.471	*r* = 0.233
	*p* = 0.001	*p* = 0.702	*p* = <0.001	*p* = 0.025

*Underlined results indicate statistical significance*.

### Hydrocephalic and Control Subgroups

**Figure 3:**
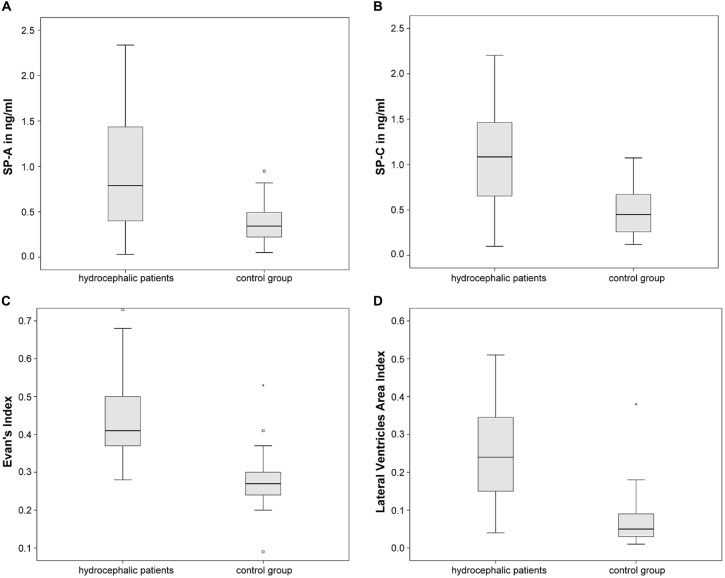
**Graphically demonstrates significant differences between the control group and all hydrocaphalic patients identified by means of ANOVA. (A,B)** Show differences in CSF concentrations of SP-A and SP-C. **(C,D)** Show differences in measures of ventricular enlargement (Evans’ Index and LVAI). Circles (°) are used to denote outliers that are farther than 1.5 interquartile ranges, yet closer than 3 interquartile ranges away from the nearer edge of the box. Little stars (*) indicate outliers that are farther than 3 interquartile ranges away from the nearer edge of the box.

CSF-SP concentrations as well as the two dilatation indices are being compared. ANOVA revealed statistically significant differences regarding CSF-SP concentrations of SP-A (*p* < 0.001) and SP-C (*p* < 0.001) between hydrocephalic patients and control subjects. The findings are graphically summarized in Figures 3A,B. No significant differences were found for SP-B and SP-D, although a trend was delineable for SP-D (*p* = 0.087).

Evans’ Index and LVAI showed statistically significant differences between hydrocephalic patients and control subjects by means of ANOVA (both *p* < 0.001), Figures 3C,D summarize the findings graphically.

### Different Hydrocephalic Subsets—Acute Hydrocephalic Conditions (AQS Plus AH) and (Chronic) NPH vs. Controls

Table 5 shows the comparative descriptive statistics of the acute hydrocephalic conditions subset vs. the NPH subset. For reasons of better comparability control values were also included in Table 5.

**Table 4:** **Comparison of CSF-SP levels, Evans’ Index and LVAI between hydrocephalic patients and controls**.

	Hydrocephalic patients (*n* = 55)		Control group (*n* = 41)	
	Mean ± SD	Range	Mean ± SD	Range
SP-A (ng/ml)	0.93 ± 0.64	0.03−2.34	0.37 ± 0.21	0.05−0.95
SP-B (ng/ml)	0.14 ± 0.33	0.00−1.41	0.24 ± 0.53	0.00−1.92
SP-C (ng/ml)	1.22 ± 0.88	0.10−5.18	0.49 ± 0.26	0.12−1.07
SP-D (ng/ml)	8.16 ± 6.74	0.00−38.03	6.20 ± 2.04	2.81−9.85
Evans’ Index	0.43 ± 0.09	0.28−0.73	0.28 ± 0.07	0.09−0.53
LVAI	0.26 ± 0.14	0.04−0.79	0.07 ± 0.07	0.01−0.38

**Table 5:** **Comparison of CSF-SP levels, Evans’ Index and LVAI between hydrocephalic subsets—acute hydrocephalic conditions (AQS plus AH) and NPH vs. the control group**.

	Acute hydrocephalic conditions (*n* = 36)		Control group (*n* = 41)		NPH (*n* = 19)	
	Mean ± SD	Range	Mean ± SD	Range	Mean ± SD	Range
SP-A (ng/ml)	1.05 ± 0.69	0.03−2.34	0.37 ± 0.21	0.05−0.95	0.69 ± 0.45	0.17−1.76
SP-B (ng/ml)	0.10 ± 0.29	0.00−1.20	0.24 ± 0.53	0.00−1.92	0.22 ± 0.39	0.00−1.41
SP-C (ng/ml)	1.45 ± 0.99	0.10−5.18	0.49 ± 0.26	0.12−1.07	0.76 ± 0.34	0.13−1.46
SP-D (ng/ml)	9.10 ± 7.75	0.00−38.03	6.20 ± 2.04	2.81−9.85	6.38 ± 3.83	0.00−13.98
Evans’ Index	0.45 ± 0.11	0.28−0.73	0.28 ± 0.07	0.09−0.53	0.41 ± 0.05	0.33−0.51
LVAI	0.28 ± 0.16	0.04−0.79	0.07 ± 0.07	0.01−0.38	0.23 ± 0.09	0.09−0.38

**Figure 4:**
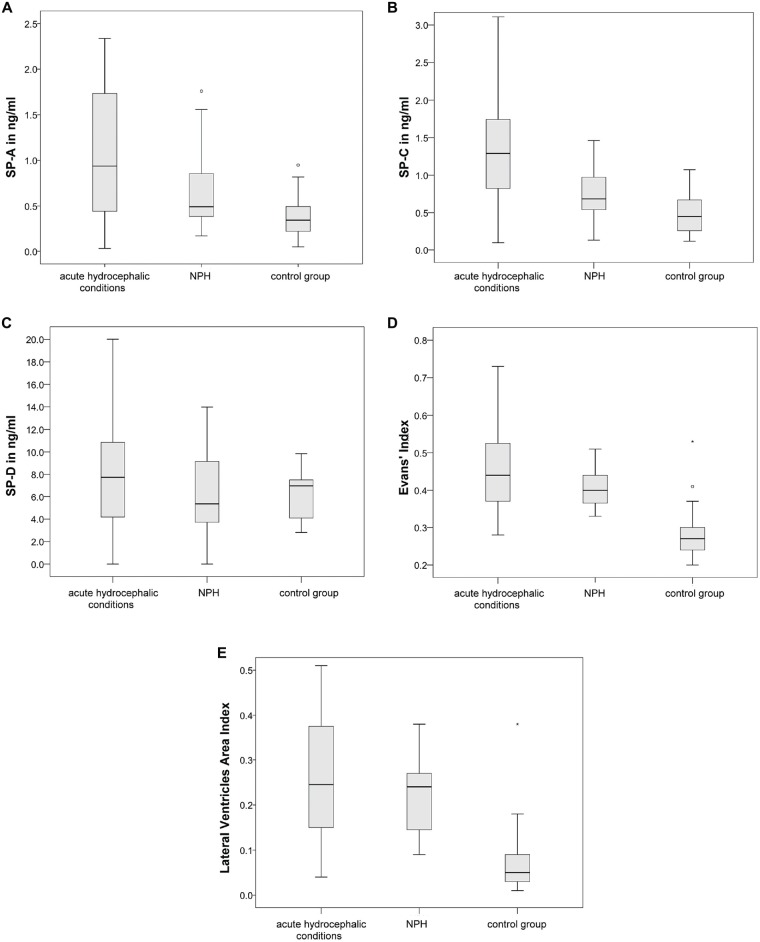
**(A–E)** Graphically demonstrates significant differences between the control group, the acute hydrocephalic conditions subset (composed of acute hydrocephalus (AH) and aqueductal stenosis (AQS) patients) and the normal pressure hydrocephalus (NPH) subset identified by means of ANOVA. **(A–C)** Show differences in CSF concentrations of SP-A, SP-C and SP-D. **(D,E)** Show differences in measures of ventricular enlargement (Evans’ Index and LVAI). Circles (°) are used to denote outliers that are farther than 1.5 interquartile ranges, yet closer than 3 interquartile ranges away from the nearer edge of the box. Little stars (*) indicate outliers that are farther than 3 interquartile ranges away from the nearer edge of the box.

ANOVA, comparing the acute hydrocephalic conditions subset, the NPH subset and the control group as three equal groups regarding CSF-SP concentrations and measures of ventricular enlargement revealed the following results: SP-A, SP-C, SP-D, Evans’ Index and LVAI, but not SP-B were significantly different between the groups (SP-A: *p* < 0.001, SP-C: *p* < 0.001, SP-D: *p* = 0.046, Evans’ Index: *p* < 0.001, LVAI: *p* < 0.001). Figures 4A–C graphically demonstrate differences in CSF-SP levels between the investigated subsets, Figures 4D,E show differences regarding Evans’ Index and LVAI.

Dunnett’s test gave the following results: values of SP-A (*p* < 0.001), SP-C (*p* < 0.001), SP-D (*p* = 0.040), Evans’ Index (*p* < 0.001) and LVAI (*p* < 0.001) were different in the acute hydrocephalic conditions subset compared to the control group. Regarding the NPH subset, Dunnett’s test showed the following results: significant differences in comparison to the control group were identified for Evans’ Index (*p* < 0.001) and LVAI (*p* < 0.001). SP-A showed a clear trend, but the difference in SP-A levels between NPH subset and control group did not reach statistical significance (*p* = 0.052).

It was demonstrated that both hydrocephalic subsets, the acute hydrocephalic conditions subset and the NPH subset, exhibit significant ventricular enlargement compared to the control group. However, only the acute hydrocephalic conditions subset revealed significant differences in CSF-SP concentrations compared to controls. To further investigate the association between enlargement of the inner CSF spaces and SP-CSF concentrations in both hydrocephalic subsets, ANOVA (regarding measures of ventricular enlargement and CSF-SP concentrations) was performed including only both hydrocephalic subsets.

**Figure 5:**
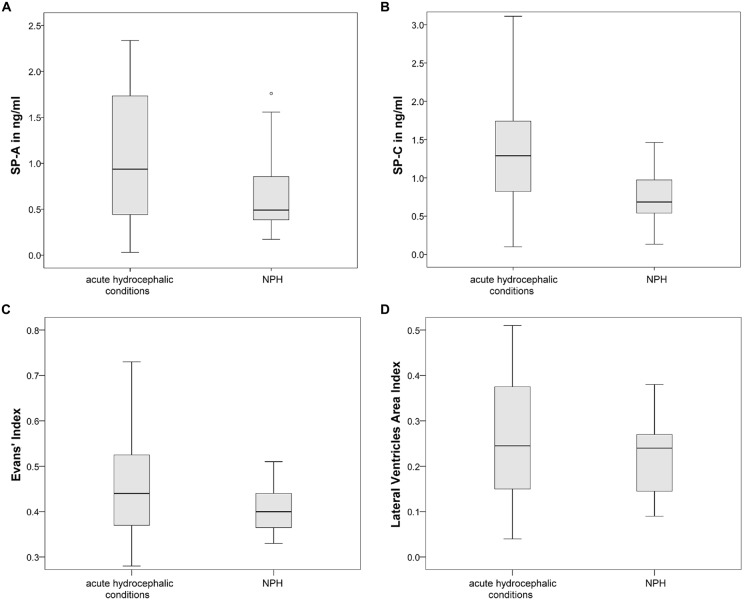
**Graphically demonstrates significant differences between the acute hydrocephalic conditions subset (composed of AH and AQS patients) and the NPH subset identified by means of ANOVA. (A,B)** Show differences in CSF concentrations of SP-A and SP-C. **(C,D)** Show that there are no significant differences in measures of ventricular enlargement (Evans’ Index and LVAI) between both hydrocephalic subsets. Circles (°) are used to denote outliers that are farther than 1.5 interquartile ranges, yet closer than 3 interquartile ranges away from the nearer edge of the box.

As expected, measures of ventricular dilatation did not reveal statistically significant differences between the acute hydrocephalic conditions subset and the NPH subset. In contrary, CSF-SP concentrations were significantly different in the NPH subset compared to the acute hydrocephalic conditions subset (SP-A: *p* = 0.046, SP-C: *p* = 0.008). Figures 5A,B graphically demonstrate differences in CSF-SP concentrations between the NPH subset and the acute hydrocephalic conditions subset, Figures 5C,D demonstrate comparable values (no differences) of ventricular enlargement in both subsets.

Additionally, correlation analysis of ventricular dilatation indices and CSF-SP concentrations was performed in both hydrocephalic subsets (the NPH subset and the acute hydrocephalic conditions subset). Spearman correlation analysis yielded the following results: in both subsets there was a highly significant correlation between SP-A and SP-C concentrations with both dilatation indices. The acute subset even showed a significant correlation of SP-D with both Evans’ Index and LVAI, which was not detectable in the NPH subset.

In the acute hydrocephalic conditions subset values were as follows: SP-A with Evans’ Index (*r* = 0.458, *p* < 0.001) and LVAI (*r* = 0.433, *p* = 0.001), SP-C with Evans’ Index (*r* = 0.457, *p* < 0.001) and LVAI (*r* = 0.519, *p* = 0.001) and SP-D with Evans’ Index (*r* = 0.241, *p* = 0.038) and LVAI (*r* = 0.271, *p* = 0.019).

In the NPH subset values were as follows: SP-A with Evans’ Index (*r* = 0.438, *p* = 0.001) and LVAI (*r* = 0.326, *p* = 0.013) and SP-C with Evans’ Index (*r* = 0.365, *p* < 0.007) and LVAI (*r* = 0.315, *p* = 0.017).

**Figure 6:**
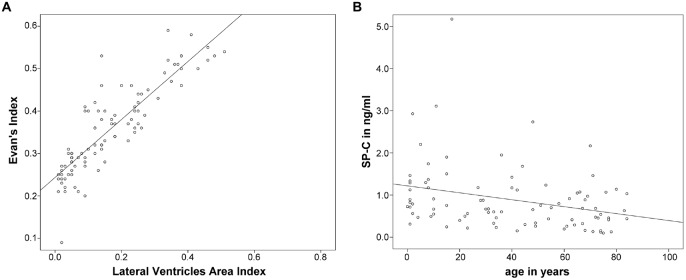
**(A,B)** Graphically demonstrates the idientfied correlation between both measures of ventricular enlargement (Evans’ Index and LVAI) as well as the inverse correlation between SP-C in CSF and age.

Finally, the relation of both dilatation indices was examined more closely. Evans’ Index correlated well with LVAI (*r* = 0.884, *p* < 0.0001) in the overall population, the association between both parameters is graphically demonstrated in Figure 6A. Analyzing the data exclusively from hydrocephalic patients the correlation was as follows: *r* = −0.357, *p* = 0.011. Analyzing the data exclusively from the control group the correlation was as follows: *r* = −0.327, *p* = 0.039.

As an additional finding, SP-C showed a clear inverse correlation with age when analyzing data from all investigated subjects (*r* = −0.343, *p* = 0.001; Figure 6B).

## Discussion

Our study is the first to report the relation between dilatation of the brain ventricles and SP concentration in CSF.

According to the literature, SPs fulfill a variety of organ-specific functions in various extrapulmonary tissues (Bräuer et al., 2007a,b; Snegovskikh et al., 2011). Besides their important role as specialized antibodies of the innate immune system (Palaniyar, 2010), SPs support the stability and rheology of fluids at interfaces in the body (Schicht et al., 2015).

Concerning this matter it is of great interest that SPs have been identified as inherent proteins of the CNS, synthesized and secreted by choroid plexus and ependymal cells, which are also the major sites of CSF production (Schob et al., 2013, 2016a).

Also in some, but not all hydrocephalic conditions SPs were shown to be significantly elevated in the CSF of patients compared to normal subjects (Schob et al., 2016b). In detail it was demonstrated that SP-A and SP-C levels (in contrary to SP-B and SP-D) in the CSF were significantly higher in patients suffering from rather acute hydrocephalic conditions, but not in patients suffering from NPH, who also exhibit significant enlargement of the inner CSF spaces. This raises the question after a possible relationship between the phenomena of elevated CSF-SP levels and ventricular enlargement.

Dilatation of the brain ventricles in adults, being the hallmark of hydrocephalic conditions, can at best be diagnosed and monitored by use of cross sectional imaging. Different approaches and indices for quantification of ventricular enlargement exist. Ventricular dilatation is most accurately estimated using volumetric, three dimensional approaches (Toma et al., 2011; Ishii et al., 2013; Ragan et al., 2015).

However, to reliably perform volumetric measurements isovoxel 3D datasets have to be acquired in MRI (Chaarani et al., 2014). Unfortunately, 3D datasets are not included in most MRI protocols intended for clinical routine purposes in our hospital, since they are relatively time consuming and prone to motion artifacts. Patients suffering from neurological conditions often cannot lay still during a long lasting MRI investigation for they often present with movement and shaking troubles. Therefore, 3D datasets were not available in most cases for our study. As a consequence of that we chose to evaluate ventricular dilatation using an existing, uni-dimensional approach (Evans’ Index) as well as a self-designed, hereby introduced two-dimensional approach (LVAI).

The Evans’ Index is an easy, rapid and robust linear method not requiring any specialized software (Ambarki et al., 2010). It displays the ratio of maximum width of the frontal horns of the lateral ventricles and the maximal diameter of the skull measured in the same axial section in a CT or MRI scan. However, Toma et al. (2011) discussed that Evans’ Index is not an ideal method for estimation of ventricular enlargement, for it can vary significantly in a patient with NPH, depending on the level of the brain scan image at which the frontal horns and maximal inner skull diameters are measured. Nevertheless, Evans’ Index is still used in clinical routine (Lieb et al., 2015) and has been shown to correlate excellently with ventricular volume (Reinard et al., 2015). Despite the aforementioned, Evans’ Index does not account for asymmetrical or uneven enlargement of the lateral ventricles (Ragan et al., 2015).

To reduce possible bias in the evaluation of ventricular enlargement secondary to asymmetrical or uneven ventricular dilatation, we designed the two-dimensional LVAI, which shares common features with the (like Evan’s Index uni-dimensional) frontal occipital horn ratio (O’Hayon et al., 1998). As shown by Chaarani et al. (2013), two-dimensional estimations of the lateral ventricles can be used reliably to assess ventricular volume, if 3D images are not available for reasons discussed above.

Evans’ Index and LVAI revealed an excellent correlation in our study, indicating that both reflect ventricular enlargement. This is further underlined by the results of ANOVA, showing that Evans’ Index and LVAI were significantly different in hydrocephalus patients compared to the healthy control group.

In the overall collective, SP-A and SP-C revealed clear correlations with Evans’ Index, which corresponds well to the findings of our previously published study (Schob et al., 2016b). As anticipated, SP-A and SP-C also correlated well with the LVAI. In fact, the correlation of SP-C and LVAI revealed to be even superior compared to Evans’ Index. Also, SP-D showed a moderate correlation with LVAI, but not with Evans’ Index. This might indicate that LVAI indeed measures additional features of ventricular enlargement, which are not accounted for by Evans’ Index.

As mentioned above, one of the major findings of our previous study (Schob et al., 2016b) was a significant increase of SP-A and SP-C in diseases with ventricular enlargement—with the exception of NPH. This challenged the formulated hypothesis that ventricular enlargement as such is associated with increased concentrations of these two SPs, and may instead be limited to acute events. Yet the extent of ventricular enlargement in these cases had not been determined. Analyzing the current data, we found comparably increased ventricular width in both acute and NPH subsets compared to the control population. Again, SP-A and SP-C showed significantly elevated levels in the acute hydrocephalic conditions, but no significant elevation in NPH (whereas SP-A showed a clear trend of increase in NPH vs. the control group). Yet, the measured SP concentrations correlated well with both dilatation indices in every subset, including NPH. This corroborates an indeed existing general relation between ventricular width and SP-concentration, even if the mere concentrations do not reflect significant alterations in case of NPH. It may be speculated that, as part of probable compensatory mechanisms due to a prolonged course of the disease, the SP-concentrations are not as notably elevated in NPH, resulting in the delineable trend, but not statistical significance.

Surprisingly, SP-C revealed a clear inverse correlation with age in our present study. This association was identifiable when calculating the Spearman correlation coefficient including hydrocephalus patients only, including the control group only and including all investigated individuals. This might indicate that SP-C generally decreases in the CSF with growing age.

## Conclusion

The present study confirmed a clear correlation between SP-A and SP-C in the CSF with enlargement of the inner CSF spaces. Furthermore, a moderate correlation between SP-D in CSF with ventricular enlargement was identified by using a more accurate, area measurement based approach compared to the simple, linear approach. This possibly indicates that SP-D is also differently regulated in hydrocephalic conditions, but in a more subtle manner than SP-A and SP-C. In conclusion, SPs clearly play an important role for CSF rheology. CSF rheology is profoundly altered in hydrocephalic diseases, however, diagnosis and therapy of hydrocephalic conditions are still almost exclusively based on ventricular enlargement. Until now it was unclear, whether the stage of the disease, as represented by the extent of ventricular dilatation, is somehow related to the changes of SP levels in the CSF. Our study is the first to provide evidence that aggravated ventriculomegaly is accompanied by enhanced changes of rheologically active compounds in the CSF and therefore introduces completely new aspects for hydrocephalus testing and conservative therapeutic approaches.

## Author Contributions

SS performed the experiments and wrote the article. AW performed experiments. JD, CR, MPirlich wrote the article and performed statistical analysis. PV, AS and K-TH wrote the article. UQ and MPreuß designed the experimental setting and wrote the article. UQ and MPreuß contributed equally to the article.

## Conflict of Interest Statement

The authors declare that the research was conducted in the absence of any commercial or financial relationships that could be construed as a potential conflict of interest.
